# Distinct Distribution of Ectopically Expressed Histone Variants H2A.Bbd and MacroH2A in Open and Closed Chromatin Domains

**DOI:** 10.1371/journal.pone.0047157

**Published:** 2012-10-30

**Authors:** Elena S. Ioudinkova, Ana Barat, Andrey Pichugin, Elena Markova, Ilya Sklyar, Iryna Pirozhkova, Chloe Robin, Marc Lipinski, Vasily Ogryzko, Yegor S. Vassetzky, Sergey V. Razin

**Affiliations:** 1 Institute of Gene Biology, Russian Academy of Sciences, Moscow, Russia; 2 CNRS UMR 8126, Univ. Paris-Sud 11, Institut de cancérologie Gustave Roussy, Villejuif, France; 3 LIA1066, Laboratoire Franco-Russe de recherches en oncologie, Villejuif, France; 4 The Centre for Scientific Computing & Complex Systems Modelling (SCI-SYM), School of Computing, Dublin City University, Dublin, Ireland; Université Paris-Diderot, France

## Abstract

**Background:**

It becomes increasingly evident that nuclesomes are far from being identical to each other. This nucleosome diversity is due partially to the existence of histone variants encoded by separate genes. Among the known histone variants the less characterized are H2A.Bbd and different forms of macroH2A. This is especially true in the case of H2A.Bbd as there are still no commercially available antibodies specific to H2A.Bbd that can be used for chromatin immunoprecipitation (ChIP).

**Methods:**

We have generated HeLa S3 cell lines stably expressing epitope-tagged versions of macroH2A1.1, H2A.Bbd or canonical H2A and analyzed genomic distribution of the tagged histones using ChIP-on-chip technique.

**Results:**

The presence of histone H2A variants macroH2A1.1 and H2A.Bbd has been analyzed in the chromatin of several segments of human chromosomes 11, 16 and X that have been chosen for their different gene densities and chromatin status. Chromatin immunoprecipitation (ChIP) followed by hybridization with custom NimbleGene genomic microarrays demonstrated that in open chromatin domains containing tissue-specific along with housekeeping genes, the H2A.Bbd variant was preferentially associated with the body of a subset of transcribed genes. The macroH2A1.1 variant was virtually absent from some genes and underrepresented in others. In contrast, in closed chromatin domains which contain only tissue-specific genes inactive in HeLa S3 cells, both macroH2A1.1 and H2A.Bbd histone variants were present and often colocalized.

**Conclusions:**

Genomic distribution of macro H2A and H2A.Bbd does not follow any simple rule and is drastically different in open and closed genomic domains.

## Introduction

When the nucleosome particles were discovered [1], [2] they were considered uniform basic blocks of a chromatin fiber. Since this time it became evident that nucleosomes are far from being identical [3], [4]. This nucleosome diversity is due partially to the existence of histone variants. The variant forms of histones encoded by separate genes were discovered long ago but their functional significance remained obscure until recently [5], [6], [7]. One of the best characterized histone variants is H3.3, which is enriched in nucleosomes of actively transcribed genes [8]. The impact of other histone variants on transcription is less clear. Thus, some data link histone H2A.Z with actively transcribed genomic regions [9], while the other suggest that this histone participates in formation of inactive heterochromatin [10]. Histones macroH2A and H2A.Bbd are among the histone variants whose function is the most obscure. It was reported that macro H2A is preferentially concentrated in the non-active copy of X chromosome (Barr body) in female cells of mammals [11], while the H2A.Bbd (Barr body deficient) is excluded from this chromosome [12]. It was thus proposed that these histones participate in formation of repressed and active chromatin, respectively. Indeed, incorporation of H2A.Bbd into nucleosomal core had a negative effect on the stability of the corresponding nucleosomes [13], [14] and 30 nm chromatin fibers [15]. Studies of several genomic areas supported the idea that macroH2A was involved in gene silencing [16], [17], [18], [19]. However, while macroH2A was found to be preferentially associated with repressed genes, about 12% of genes associated with macroH2A were found to be transcribed [20]. Thus its effect on transcription may vary and depends on chromatin context. H2A.Bbd was studied mostly in *in vitro* experiments, which demonstrated that incorporation of this histone variant into core particle facilitated transcription [15], [21]. In double labeling experiments, the patterns of nuclei and metaphase chromosome staining with antibodies recognizing H2A.Bbd and H4 acetylated at lysine 12 were found to be very similar suggesting localization of H2A.Bbd in active chromatin [12].

Expression of H2A.Bbd is almost exclusively confined to spermiogenic fractions of mammalian testis and nucleosomal chromatin fraction of mature human sperm [22], after the shut-down of transcription [23]. The ectopically expressed non-tagged version of the protein is enriched at the periphery of chromocenters [22]. At the same time, aberrant activation of testis-specific genes may lead to malignant transformation of cells [24].

In order to obtain more information about possible function of H2A.Bbd and macroH2A in normal and pathogenic situations, we have now mapped the sites of preferential location of H2A.Bbd and macroH2A1.1 in differently organized chromatin domains in human epithelial HeLa S3 cells ectopically expressing tagged histones H2A, macroH2A1.1 or H2A.Bbd. Two genomic regions were analyzed most thoroughly. One of them spans 700 Kb on the short arm of chromosome 11 and harbors tissue-specific olfactory receptor and beta-globin genes. These genes are not expressed in HeLa S3 cells. The domain of beta-globin genes, inserted into a larger cluster of olfactory receptor (OR) genes, represents the so-called “closed chromatin domain”, which is organized in compact (DNAse I resistant) chromatin in cells where globin genes are not expressed [25], [26]. The same is true for the OR genes.

The second domain studied occupies the telomeric end (500 Kb) of the short arm of the chromosome 11. This domain harbors many house-keeping genes along with a tissue-specific alpha-globin gene cluster [26], [27], [28]. Due to this reason, the whole 500 Kb region maintains an open (DNase I sensitive) chromatin configuration in cells of different lineages. The borders of a tissue specific domain of alpha-globin genes can be defined only functionally. The domains of this type are referred to as “open” or “functional” chromatin domains [25], [26].

We have studied whether the above-discussed differences of the two chromatin domains correlated with different patterns of H2A.Bbd and macroH2A1.1 deposition into these genomic areas. The results of the analysis suggest that gene silencing does not always correlate with deposition of macroH2A1.1 and that H2A.Bbd is present in both active and repressed chromatin domains. The significance of these observations was further corroborated by the analysis of macroH2A1.1 and H2A.Bbd distribution in 1000 Kb-long fragment of Chromosome 11, harboring several widely-expressed genes including *CCND1* and in a 500 Kb-long segment of Chromosome X, harboring a part of the *DMD* gene.

## Results

### H2A.Bbd and macroH2A1.1 show appropriate subcellular localization in transfected HeLa cells

We first examined subnuclear localization of endogenous macroH2A1 in HeLa S3 cells and then compared it to that in HeLa S3 stably transfected with the pOZ vector expressing epitope-tagged macroH2A1.1 The macroH2A1.1 distribution patterns in normal and transfected HeLa S3 cells were quite similar (Figure S1), cells with higher staining intensity were observed in the transfected cell line (Figure S1b, marked by arrows), but macroH2A1 distribution pattern was similar in these cell lines as compared to the control. Importantly, the distribution of macro H2A.1 in normal and transfected HeLa S3 cells was strictly nuclear and distribution of H2A.1 reflected the distribution of DNA as determined by measuring the fluorescent intensities for 3 nuclei using Zeiss LSM Image (see the graphs under the images in Figure S1a,b). The distribution of the epitope-tagged macroH2A.1.1 revealed by staining with antibodies against FLAG was quite similar to the distribution of macroH2A1.1 revealed by staining with antibodies against this histone variant (Figure S1c).

**Figure 1:**
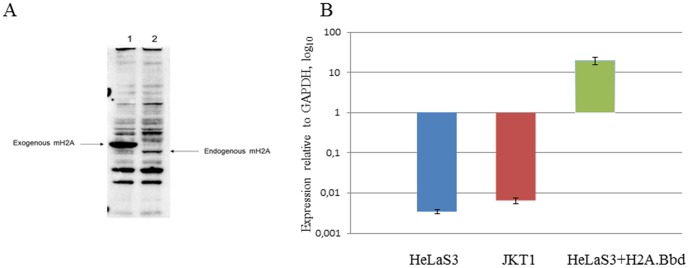
Expression levels of ectipically expressed macroH2A1.1 and H2ABbd. **a,** HeLa S3 cells overexpressing tagged macroH2A1.1 (1) as compared to endogenous macroH2A (2) in the control HeLa S3. Western Blot with staining with the anti- macroH2A antibodies (Active Motif); **b,** The expression of H2A.Bbd was measured using qRT-PCR in JKT1 human testicular seminoma cells, HeLa S3 and HeLaS3 ectopically expressing H2A.Bbd. Mean values are shown, error bars represent S.E.M. of 2 independent experiments.

Next we have determined the expression levels of endogenous and ectopic macroH2A1.1 in HeLa S3 cells. The detected level of ectopic expression of is more than ten-fold higher than the expression of endogenous macroH2A1.1 (Figure 1A). Interestingly, the expression of exogenous tagged macroH2A1.1 leads to inhibition of the endogenous macroH2A suggesting a feedback mechanisms in regulation of macroH2A synthesis.

**Figure 2:**
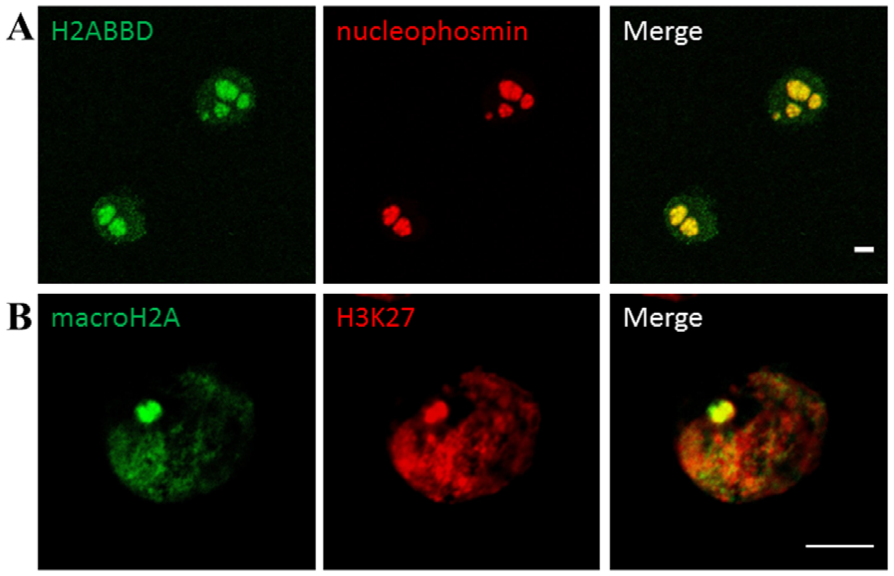
Subnuclear localisation of macroH2A and H2A.Bbd in HeLa S3cells. HeLa S3 cells stably transfected with either the pOZ -macroH2A1.1 (**a**) or pOZ- H2A.Bbd (**b**) plasmid and stained with antibodies against FLAG (green) and H3k27Me3 or nucleophosmin (red). **a**: macroH2A1.1 is preferentially localised to a single region in interphase HeLa cells and coinsides with H3k27Me3 staining (red). **b**: H2A.Bbd has a punctate nuclear staining with an exclusion zone (marked by a white arrow) and a strong perinucleolar staining (nucleophosmin, red). The figure shows representative confocal sections. Scale bar: 5 µm.

Upon analysis at higher magnification under confocal microscope, a large stained focus of recombinant H2A1.1 was observed. It was also stained with the antibodies against H3K27Me3, typical for the inactive X chromosome (Figure 2a).

We could not compare the expression of the epitope-tagged H2A.Bbd to that of its endogenous variant, as the commercially available antibodies against recombinant H2A.Bbd do not recognize endogenous H2A.Bbd in HeLa cells, therefore we used qRT-PCR to determine its expression in JKT1 testicular seminoma cell line, HeLa S3 and HeLa S3 ectopically expressing H2A.Bbd (Figure 1B). We have detected a low level of expression of H2A.Bbd both in HeLa and in JKT1, suggesting that it lay be expressed in the context of cancer. The level of ectopically expressed H2Bbd was significantly higher than that in the control.

Stable transfection of HeLa S3 cells with pOZ- H2A.Bbd showed even localization of the epiotope-tagged H2A.Bbd within the nucleus with strong perinucleolar enrichment (Figure 2b). Similar localization patterns of H2A.Bbd have been previously observed in somatic female human cells [29] and in embryonic stem cells [30].

### Analysis of the distribution of H2A.Bbd and macroH2A1.1 within selected regions of human chromosomes 11 and 16

Nucleosomes containing tagged versions of H2A.Bbd and macroH2A1.1 were isolated. DNA was extracted and used as a probe for hybridization with custom NimbleGene genomic microarrays covering the telomeric end of the short arm of human chromosome 16 (positions 1–500,000) and a 700 Kb-long fragment of the short arm of human chromosome 11 (positions 4,900,000–5,600,000). The microarray was prepared with a median probe spacing of 50 bp. Both genomic regions are included in the Pilot Encode project, making data on histone modifications available via the Integrated Genome Browser [31].

The distributions of H2A.Bbd and macroH2A1.1 were compared to that of the major H2A form, similarly tagged. To this aim, DNA associated with tagged H2A was isolated in the same way as with tagged H2A.Bbd and macroH2A1.1. The DNA samples associated with tagged H2A were used for reference hybridization in both experimental sets.

The results of hybridization with microarrays were analyzed using the ACME (Algorithm for Capturing Microarray Enrichment) program [32], [33] as described in Materials and methods. The program identifies the probes representing the genomic region under study and scans this region using a window size which can be user-defined. We have chosen a window size of 400 bp because it will cover DNA fragments containing up to two nucleosomes. The p-values assessing a possible association of DNA with either H2A.Bbd or macroH2A1.1 were calculated and represented as graphs along the chromosomic regions studied.

**Figure 3:**
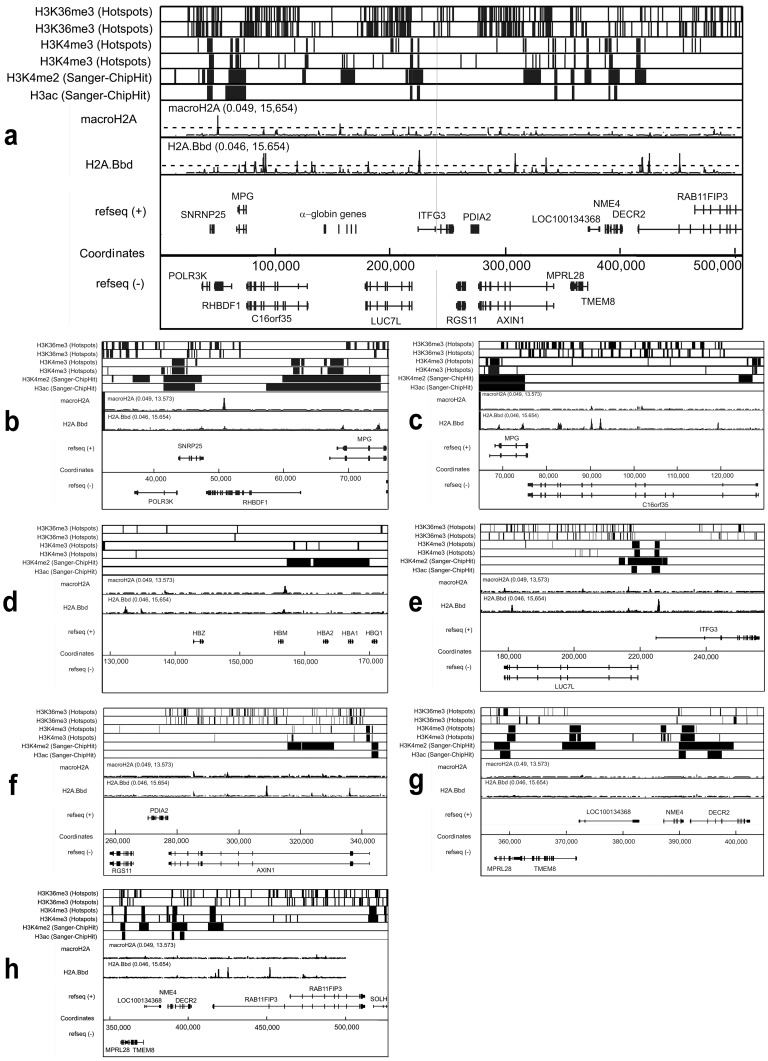
Distribution of sites of preferential location of macroH2A1.1 and H2A.Bbd within the telomeric end of the short arm of human chromosome 16. (**a**) Low magnification picture showing the whole area under study. (**b–f**) High magnification (zoomed in) pictures showing consecutive sections of the area under study. Distribution of histone H3 tri-methylated at lysine 36 (H3K36me3), histone H3 tri-methylated at lysine 4 (H3K4me3), histone H3 di-methylated at lysine 4 (H3K4me2) and panacetylated histone H3 (H3ac) is presented according to the Pilot Encode datasets. Positions of genes are shown according to the UCSC genome browser (assembly HG18). For H3K36me3 and H3K4me3 two independent sets of data are shown. The values in brackets above the graphs showing distribution of macroH2A1.1 and H2A.Bbd represent the range of the transformed signal, (range of −log_10_(p-values) of the ACME p-values). The black broken lines in the sections showing distribution of macroH2A1.1 and H2A.Bbd indicate the significance level (−log_10_(p-value = 4)).

**Figure 4:**
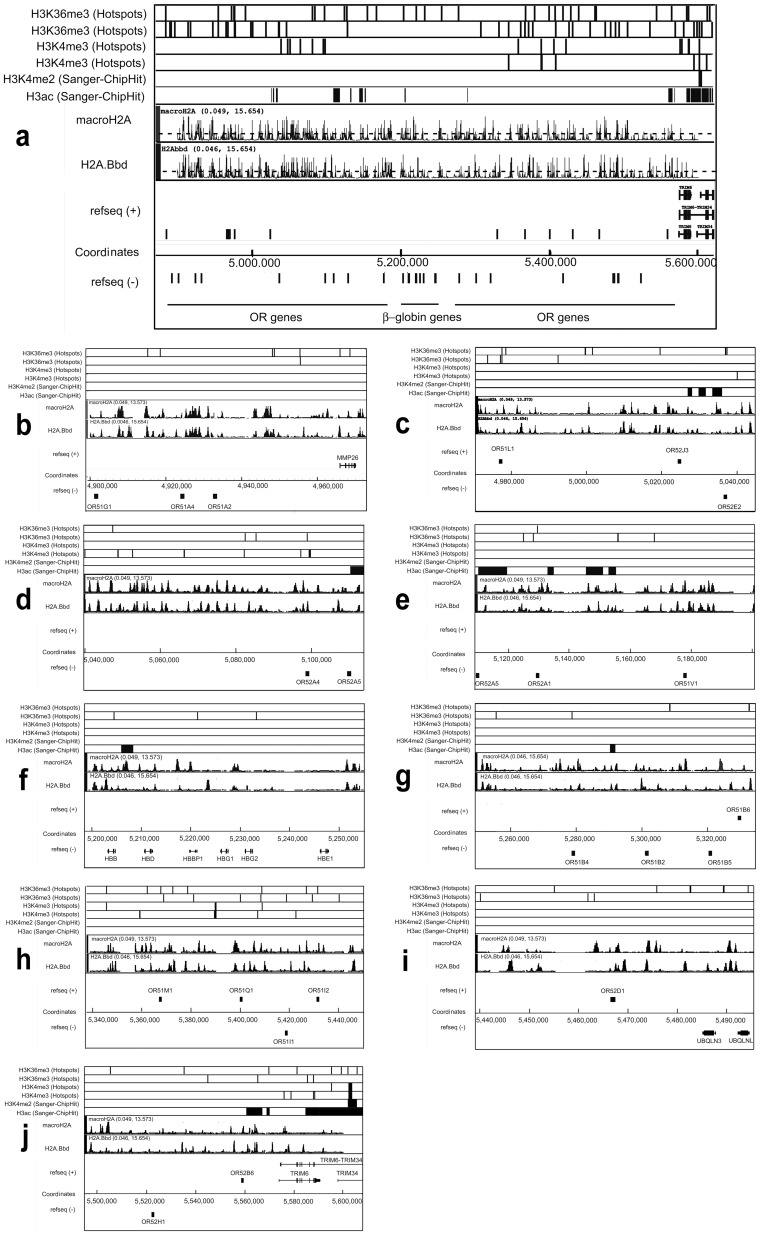
Distribution of sites of preferential deposition of macroH2A1.1 and H2A.Bbd within the OR - β-globin gene domain in human chromosome 11. (**a**) Low magnification picture showing the whole area under study. (**b–f**) High magnification (zoomed in) pictures showing consecutive sections of the area under study. All designations are the same as in Figure 3.

Results obtained for the two selected regions on chromosomes 16 and 11 are shown in Figures 3 and 4, respectively. The known distributions of histone H3 tri-methylated at lysine 36 (H3K36me3) or at lysine 4 (H3K4me3), histone H3 di-methylated at lysine 4 (H3K4me2) and panacetylated histone H3 (H3ac) are reported according to the Pilot Encode datasets in the upper part of the figures. In the middle part, the distribution of sites of preferential localization of macroH2A1.1 and H2A.Bbd is presented. Alignment with gene positions is shown in the lower part of the figures.

From the analysis of the data presented in Figure 3, it is clear that in the 500 Kb rsegment of chromosome 16p which contains the alpha-globin gene domain, there are very few regions with macroH2A1.1-containing nucleosomes. This contrasts with the larger number of regions with H2A.Bbd-containing nucleosomes. Most of the H2A.Bbd peaks were located within bodies of transcribed genes, and not in the vicinity of transcriptional start sites (TSS) (Table 1). It is of note that alpha-globin genes that are not expressed in HeLa cells were not preferentially associated with macroH2A1.1 (Figure 3d). In the analysis of the chromosome 11p fragment reported in Figure 4, peaks corresponding to both H2A.Bbd and macroH2A1.1 were found scattered all over the region. As seen in the enlarged images shown in Fig. 4b–j, regions enriched in H2A.Bbd and macroH2A1.1 often mapped to the same genomic locations. It can also be seen that most of the sites enriched in H2A.Bbd and macroH2A1.1 lied within intergenic regions. This, however, may simply reflect the fact that genes occupy relatively small portions of this 700 Kb-long chromosomal fragment. At the centromeric end of the region, H2A.Bbd nevertheless appeared preferentially associated with the TRIM6 gene (Figure 4j).

**Table 1:** Presence of H2A.Bbd and macroH2A1.1 loading sites within gene bodies and promoter areas (2500 to +1000 and from −5000 to +1000) of genes present in the selected areas of the chromosome 11 and the chromosome 16.

Gene (name)	Transcription status Max(log2(Intensity_probes))	Number of H2A.Bbd peaks in the body of the gene	Number of H2A.Bbd peaks located close to promoter (from −2500 to +1000)	Number of H2A.Bbd peaks located close to promoter (from −5000 to +1000)	Number of macro-H2A peaks in the body of the genes	Number of macro- H2A. peaks located close to promoter (from +1000 to −2500)	Number of macro- H2A. peaks located close to promoter (from +1000 to −5000)	Details
**CHR 11**								
OR51G1	-	0	1	1	0	1	1	1 col
OR51A4	-	0	3	5	0	3	5	5 col
OR51A2	-	0	0	0	0	0	0	
MMP26	3.359	0	1	1	1	0	1	
OR51L1	-	0	0	1	0	1	2	
OR52J3	-	0	1	1	0	2	2	
OR52E2	-	0	1	2	0	0	1	1 col
OR52A4	-	1	1	1	0	1	1	
OR52A5	-	0	1	1	0	1	1	1 col
OR52A1	3.133	0	0	0	0	2	2	
OR51V1	-	0	2	3	1	0	1	1 col
HBB	3.842	0	0	0	1	2	3	
HBD	3.156	0	1	1	0	1	2	1 col
HBBP1	-	0	1	1	1	0	0	
HBG1	4.034	0 (lack pr)	0	0	0 (lack pr)	2	2	
HBG2	3.711	0 (lack pr)	0	0	0 (lack pr)	0	0	
HBE1	3.810	0	0	1	0	0	2	
OR51B4	2.893	0	1	1	1	3	3	
OR51B2	3.519	0	0	1	0	0	0	
OR51B5	2.858	0	0	1	0	1	2	
OR51B6	2.937	0	1	0	0	0	1	
OR51M1	3.107	0	0	1	0	0	1	1 col
OR51Q1	-	0	2	2 (lack pr)	0	3	3 (lack pr)	2 col
OR51I1	3.379	0	1	1	0	0	1	
OR51I2	3.538	0	0	1	1	1	2	
OR52D1	3.399	1	1	1	0	0	1	
UBQLN3	2.902	1	1	3	0	0	2	
UBQLNL	0.828	0	3	3	0	2	2	
OR52H1	-	0	0	0	0	0	0	
OR52B6	-	0	1(lack pr)	2	1	2 (lack pr)	3	1 col
TRIM6	3.771	3	0	1	0	0	0	
**Totals**		**6**	**24**	**37**	**7**	**28**	**47**	
**CHR16**								
POLR3K	8.794	0	0	0	0	0	0	
SNRPNP25	-	0	0	0	0	0	0	
RHBDF1	6.449	0	0	0	1	0	0	
MPG	7.853	2	0	0	0	0	0	
C16orf35	6.826	4	0	1	2 (marginal)	0	0	
HBZ	3.414	0 (lack pr)	0 (lack pr)	0 (lack pr)	0 (lack pr)	0 (lack pr)	0 (lack pr)	
HBM	4.097	0	0	0	0	1	0	
HBA2	-	0 (lack pr)	0 (lack pr)	0 (lack pr)	0 (lack pr)	0 (lack pr)	0 (lack pr)	
HBA1	4.779	0 (lack pr)	0 (lack pr)	0 (lack pr)	0 (lack pr)	0 (lack pr)	0 (lack pr)	
HBQ1	3.663	0	0 (lack pr)	0 (lack pr)	0	0 (lack pr)	0 (lack pr)	
LUC7L	7.529	1	0 (lack pr)	0 (lack pr)	3(marginal)	0 (lack pr)	0 (lack pr)	
ITFG3	7.514	1	0	0	0	0	0	
RGS11	3.579	0	0	0	0	0	0	
ARHGDIG	6.143	0	0	0	0	0	0	
PDIA2	3.504	0	0	0	0	0	0	
AXIN1	6.690	3	0	0	1 (marginal)	0	0	
MRPL28	8.822	0	0	0	0	0	0	
TMEM8	7.096	0	0	0	0	0	0	
LOC1134368	-	0	0	0	0	0	0	
NME4	8.876	0	0	0	0	0	0	
DECR2	7.394	0	0	0	0	0	0	
RAB11FIP3	6.406	5	0	0	0	0	0	
RAB11FIP3	6.406	1	0	0	0	0	0	
**Totals**		**17**	**0**	**1**	**7**	**1**	**0**	

**Lack pr** - probes were not contiguous in this region.

**1 col** - a H2Abbd peak is colocalized with 1 macroH2A peak.

**Marginal** –all peaks were very low, of height just above the cut-offs, which suggest that they may be marginally significant.

The transcription rate shown was computed from raw expression data given in GDS2526 for untreated HeLa in GEO normalized using Bioconductor's rma and averaged (see Materials and Methods). The comment “lack pr” (Lack of probes) means that the corresponding regions were badly covered by tiles probes, i.e the probes were not really adjacent or tiled. In some regions, there were 100–200 bp segments not covered by probes. Designation “col” (colocalized) in the “Detail” column indicates that the peaks of macroH2A1.1 and H2A.Bbd are located in exactly the same place.

### H2A.Bbd- or macro H2A1.1-containing nucleosomes and gene expression levels

**Figure 5:**
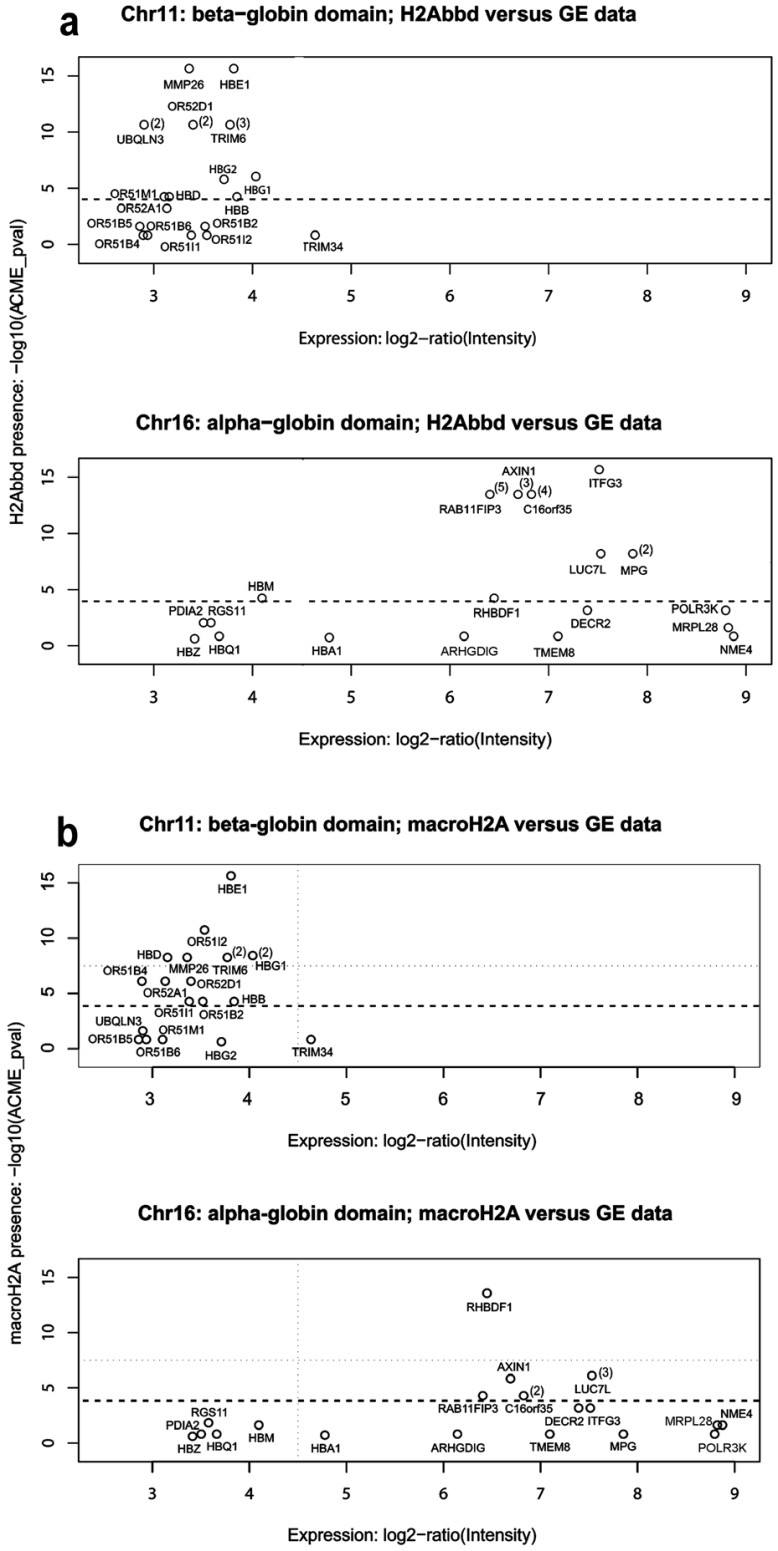
Distribution of H2A.Bbd and macroH2A1.1 on chromosomes 11 and 16. H2A.Bbd (max(−log_10_(ACME-p-values)) (**a**) and macroH2A1.1 (max (−log_10_(ACME-p-values)) (**b**) in the gene body and short upstream area [−400; TSS] versus gene expression levels is shown for genes present in the selected areas of chromosomes 11 and 16. Figures to the right of gene positions show the number of significant peaks of macro H2A (**a**) and H2A.Bbd (**b**) present within the gene body (when this number was more than 1). See the text for details.

Keeping in mind a possible role of macroH2A and H2A.Bbd in the organization of repressed and active chromatin domains, respectively, we were interested to find out whether a relationship could be described between the transcriptional level of a given gene and the presence of H2A.Bbd (or macroH2A1.1)-containing nucleosomes within the body of the gene or close to its promoter. A script was written to plot the expression level of individual genes versus max(−log_10_(ACME-p-values)) of H2A.Bbd and macroH2A1.1 associated with the respective genes. We first analyzed the transcriptional level of each gene within the selected genomic regions vs the location of H2A.Bbd (macroH2A1.1)-containing nucleosomes within the gene body plus a short upstream region (−400; TSS). Again, the results looked quite different when comparing the selected regions of chromosomes 11 and 16 (Figure 5). First of all, expression levels of genes within the selected 11p region were very low. This was not surprising since the globin and olfactory receptor genes are not expressed in HeLa cells. Although the transcription level of these genes is not exactly zero according to the databases used to extract the transcriptional data (Gene Expression Omnibus, [34]; Dataset GDS2526 [35]), it is so low that can be considered as resulting from leakage transcription. Among the genes present in the selected area of chromosome 11, *TRIM34* has the highest expression level, but corresponded to only ∼0.05% of the expression level of either *beta-actin* or *GAPDH*. We failed to observe any trend or relationship between gene transcription levels in the selected region of Chromosome 11 and the presence of either H2A.Bbd or macroH2A1.1. Many of these genes bore both H2A.Bbd and macroH2A1.1 marks.

In the selected area of Chromosome 16, transcribed genes (genes whose transcription level exceeded 1% of the GAPDH transcription level) were either associated with H2A.Bbd alone (*RAB11Fip3, AXIN1, C16orf35, IFTG3* and *MPG*) or were associated with neither macroH2A1.1 nor H2A.Bbd (*POLR3K, MPRL28* and *NME4*) (Figure 5). The only exception seemed to be the *RHBDF1* gene which was associated with macroH2A1.1. It is not clear, however, whether this gene is indeed transcribed in HeLa cells due to a discrepancy between datasets GDS3581 and GSM410912 from the GEO database.

Next, we looked for a relationship between the expression level of the genes located in the selected areas of chromosomes 11 and 16 and the presence of H2A.Bbd (macroH2A1.1) in their promoter regions (Figures S2 and S3). Again, no correlation was noted. Neither H2A.Bbd nor macroH2A1.1 were associated with the promoter regions (from −1000 to +500) of transcribed genes present in the selected region of chromosome 16. This contrasted with the promoter regions of many genes located within the selected region of chromosome 11 which contained both H2A.Bbd and macroH2A1.1.

### Distribution of macroH2A1.1 and H2A.Bbd in the *CCND1* gene region on 11q and the *DMD* gene on Xp

**Figure 6:**
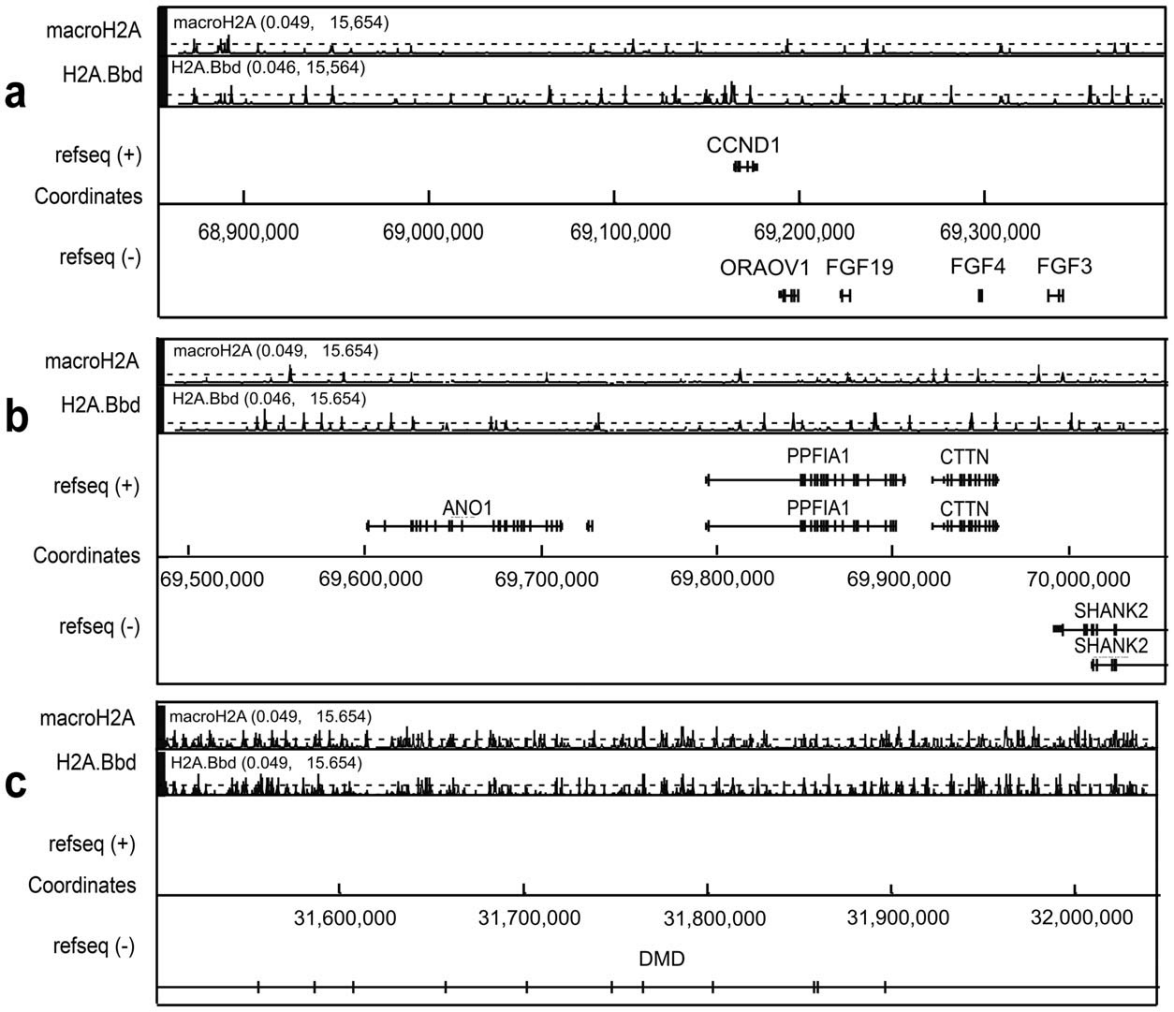
Distribution of sites of preferential location of macroH2A1.1 and H2A.Bbd within the telomeric end of the chromosome 11 (a) and the region of the X chromosome containing the DMD gene (b). The values in brackets above the graphs showing distribution of macroH2A1.1 and H2A.Bbd represent the range of the transformed signal, (range of −log_10_(p-values) of the ACME p-values). The black broken lines in the sections showing distribution of macroH2A1.1 and H2A.Bbd indicate the significance level (−log_10_(p-value = 4)).

The results described in the previous sections strongly suggested the existence of distinct patterns of macroH2A1.1 and H2A.Bbd loading in chromatin domains harboring both house-keeping and tissue-specific genes and those with tissue-specific genes only. In order to test whether this observation extended to other genomic regions, we studied the distribution of the histone variants in a 1000 Kb-long region of chromosome 11q that includes the cyclin D1 (*CCND1*) and several other genes including those coding for fibroblast growth factor (*FGF*)-related proteins. The genomic distribution of macroH2A1.1 and H2A.Bbd is presented in Figure 6. The 500 Kb-long region around the *CCND1* and neighboring genes is shown in panel “a”. The adjacent 500 Kb region on the centromeric side, which harbors additional expressed genes, is shown in panel “b”. The latter region resembles the telomeric part of chromosome 16p as it is mainly populated by gene bodies. The genomic distribution of macroH2A1.1 and H2A.Bbd in the whole 1MB genomic region also resembles that observed at the telomeric end of chromosome 16p. Indeed, there are more sites of H2A.Bbd loading than sites of macroH2A1.1 loading within both genes and intergenic region, and these sites rarely coincide with each other (Figure 5a,b and Table 2). Interestingly, the active *CCND1* gene is associated with several strong H2A.Bbd, but no macroH2A1.1 peaks (Figure 5a). From the results shown in Figure 5a,b it can be inferred that the ratio of the total length of gene bodies to intergenic regions does not determine patterns of macroH2A1.1 and H2A.Bbd loading. Indeed, gene bodies constitute less than 20% of the genomic region shown in Figure 5a vs. 60% in the next region shown in Figure 5b. Still, general patterns of macroH2A1.1 and H2A.Bbd distribution did not differ much between the two regions.

**Table 2:** The (H2A.Bbd/macroH2A1.1) ratio in 4 genomic regions studied.

Region	Ratio, ACME-p-val>5.5	Ratio, 4<ACME-p-val<5.5
ChrX, DMD	0.8789	0.8975
Chr 11, beta-globin	1.025316	1.0541
Chr 11, CCND1	2	1.2143
Chr 16, alpha-globin	4.25	1.2

Finally, we have studied the distribution of macroH2A1.1 and H2A.Bbd in a 500 Kb-long region of the short arm of chromosome X where a part of the dystrophin (*DMD*) gene is located. *DMD* is transcribed at a very low level in HeLa cells. In addition, the X- linked genes are monoallelically expressed in mammals. The patterns of loading of macroH2A1.1 and H2A.Bbd in the selected X chromosome region turned out to be very similar to those observed in the genomic domain harboring OR-β-globin genes. Indeed, as seen in Figure 5c, both macroH2A1.1 and H2A.Bbd were heavily loaded over the whole region.

## Discussion

Using stably transfected HeLa S3 cells, we have analyzed the distribution of two histone variants, H2A.Bbd and macroH2A1.1, over several regions of the human genome, well known for their different gene densities and DNA packaging in chromatin. In the selected region of chromosome 11p, small olfactory receptor and beta-globin genes occupy less than 10% of the total chromosomal fragment (Figure 4a). These genes are either not transcribed or transcribed at a very low level (leakage transcription) in HeLa cells. The whole locus was found to be depleted of active chromatin marks with the exception of a few regions associated with acetylated histone H3 (Figure 4) and thus likely to be organized in compact (DNase I-resistant) chromatin. In contrast, in the selected region of chromosome 16p, genes occupy more than 80% of the total length (Figure 3a) and more than half of them are actively transcribed (Table 1). The whole chromosomal fragment (including the non-transcribed α-globin genes) exhibits an open chromatin configuration and replicates early in cells of different lineages [36]. In HeLa cells, the locus possesses numerous active marks including H3K4 methylation at promoter areas and extended regions of H3 panacetylation (Figure 3).

In the selected fragments of chromosomes 11 and 16, the distribution of the macroH2A1.1 was characteristic. The 700 Kb-long chromosome 11 region containing inactive olfactory receptor and β-globin genes was virtually covered with macroH2A1.1, while the 500 Kb-long region on chromosome 16p which contains a number of active genes was clearly depleted of macroH2A1.1. These results are in agreement with the previously reported distribution pattern of macroH2A1.1 in IMR90 and MCF7 cells [20]. These authors had found that macroH2A1.1 was distributed in large genomic domains which, in some cases, exceeded 500 Kb. They also reported a strong negative correlation between macroH2A1.1 occupancy and gene expression, but 12% of transcriptionally active autosomal genes contained macroH2A1.1 downstream from the TSS. Thus the correlation between an active transcription status of a gene and the absence of macroH2A was convincing but not absolute.

Our own data confirm that there is no strict correlation between a gene being inactive and the presence of macroH2A.1.1 in either its body or its promoter region. Indeed, the alpha-globin genes are not expressed in HeLa cells. Yet, with the exception of *HBM*, they are not associated with macroH2A1.1 This is consistent with the fact that, in contrast to the β-globin gene domain which is nested in the larger domain of tissue-specific *OR* genes, the α-globin gene domain is located in an area rich in house-keeping genes and remains DNase I-sensitive and early-replicating in cells of different lineages [36], [37], [38]. Thus, the distribution of macroH2A1.1 appears to correlate with the chromatin configuration of these regions rather than with the actual transcription status of individual genes. This conclusion is further supported by the absence of correlation between the presence of macroH2A within gene bodies and/or promoter areas and their transcription rate (Table 1 and Figure 5).

H2A.Bbd is almost exclusively expressed in non-transcriptionally active spermiogenic cells and mature human sperm [22]. At the same time, testis-specific genes may be activated is somatic human cells and participate in their oncogenic transformation [24].

Indeed, we have detected expression of endogenous H2A.Bbd both in HeLa and JKT1, confirming its aberrant expression in tumour cells (Figure 1B). In our ChIP-on-chip experiments, the density of H2A.Bbd peaks was relatively low (Figure 3). Where the local distribution of H2A.Bbd deposition sites was analyzed within the studied chromosome 16p region, most of these sites were located in gene bodies which occupy most of the selected area (see above). We failed to find a simple relationship between the transcription rate of a gene and its level of association with H2A.Bbd (Figure 5). When present, the preferential sites of H2A.Bbd deposition were located in gene bodies rather than in the vicinity of TSS (Table 1 and Figures S1, S2). Notably, there was only one marginal site of H2A.Bbd loading within the cluster of alpha-globin genes, inactive in HeLa cells (Figure 3d).

In order to find out whether the patterns of macroH2A1.1 and H2A.Bbd distribution observed in the telomeric end of the chromosome 16q were typical for other genomic domains, we have extended our analysis to include a 1000 Kb-long region of the chromosome 11q which harbors many expressed genes including *CCND1* and *FGF* receptor-like genes. Half of the selected region harbors relatively short genes, so that the total length of gene bodies accounts for less than 20% of the total fragment. In the other half of this region, gene bodies account for approximately 60% of the total space. In spite of this difference, observed distributions of macroH2A1.1 and H2A.Bbd were similar over the whole region and resembled that observed in the telomeric end of the chromosome 16p (Figure 5a,b and Figure 3). Thus, the density of both H2A.Bbd and macroH2A1.1 peaks was relatively low and the number of H2A.Bbd peaks exceeded the number of macroH2A1.1 peaks (Table 2). As mentioned before, the H2A.Bbd peaks were not concentrated in gene bodies but were equally present in intergenic regions. Importantly, there were only few places where both macroH2A1.1 and H2A.Bbd were loaded. In conclusion, it appears that the patterns of macroH2A1.1 and H2A.Bbd loading are more related to the type of chromatin domain than to the activity of individual genes.

The most surprising result in the present study was the observation of H2A.Bbd associated with the repressed OR - β-globin gene locus on chromosome 11. The sites of H2A.Bbd association with this genomic region were as frequent as the sites of macroH2A1.1 association (Figure 4), and many of these sites colocalized with sites of macroH2A1.1 loading. Although the functional significance of H2A.Bbd loading on the OR - β-globin gene locus in chromosome 11 is not clear at the moment, it is obvious that the presence of H2A.Bbd does not interfere with the inactivated status of the locus. The phenomenon may have several explanations. The simplest one is that mechanisms ensuring long term repression of genomic domains overpower any activation effect that may be imposed by H2A.Bbd. In this regard, it may be of importance that H2A.Bbd is a relatively recent acquisition of evolution as only mammals possess this H2A variant [39]. In contrast, macroH2A is highly conserved among all vertebrates [40]. As for the general silencing mechanisms in constitutive and facultative heterochromatin, they are similar in species as distant as Humans and Drosophila [41]. The H2A.Bbd histone variant could thus mediate some fine tuning of chromatin in relation with transcriptional activity but would not be able to overcome stable silencing. H2A.Bbd may also be present in only one of the two homologous chromosomes. Interestingly, OR genes are characterized by monoallelic expression in olfactory epithelial cells [42], [43]. Thus, one allele might be associated with repressive (including macroH2A1.1), the other, with active chromatin marks (including H2A.Bbd). As ChIP analysis does not discriminate between homologous chromosomes, a superimposition of two patterns cannot be ruled out. As seen in the region of the X chromosome analyzed here (Figure 6c), the patterns of macroH2A1.1 and H2A.Bbd distribution strongly resembled those observed in the OR gene cluster on chromosome 11p. Both macroH2A1.1 and H2A.Bbd were heavily deposited over the whole region, and in numerous places their peaks colocalized with each other. Keeping in mind that X-linked genes are characterized by monoallelic expression and that the inactive copy of the X chromosome is associated with macroH2A1.1, one can easily explain the observed pattern by a superimposition of patterns for the inactive (preferential deposition of macroH2A1.1) and active (preferential deposition of H2A.Bbd) copies of the chromosome, similarly to the OR gene locus. It is also of note that *DMD* is transcribed at a very low level in HeLa cells and that X-chromosome inactivation in mammalian females is established early in development in all cells. If our interpretation is correct, then the allelic exclusion would also be established early in development, deposition of either H2A.Bbd or macroH2A1.1 marking potentially active and fully repressed alleles, respectively. The former will be activated only in cells of the appropriate lineage, both active and inactive marks remaining in cells whose alleles are not transcribed. The described results were obtained with ectopically expressed tagged variant histones, therefore it cannot be excluded that actual localization of endogenous histones in the genome differs from that observed in our studies. Further studies are necessary to confirm our observations.

## Conclusions

In HeLa S3 cells the patterns of macroH2A and H2A.Bbd loading are drastically different in open chromatin domains containing both tissue-specific and house-keeping genes and closed chromatin domains containing only tissue-specific genes non-expressed in HeLa cells. In open chromatin domains the H2A.Bbd variant is preferentially associated with the body of a subset of transcribed genes. The macroH2A variant is virtually absent from some genes and underrepresented in others. Inactive tissue-specific genes present within open chromatin domains are not preferentially associated with macro H2A1.1. Although overall H2A.Bbd is deposited in transcribed areas preferentially as compared to macroH2A1.1, there is no simple correlation between the level of gene transcription and the abundance of H2A.Bbd. In closed chromatin domains both macroH2A and H2A.Bbd histone variants are present and often colocalize. This is true in gene bodies as well as in intergenic regions.

### Note added in proof

While this MS was in revision, a paper describing genome-wide distribution of ectopcally expressed H2A.Bbd appeared [44]. The authors conclude that H2A.Bbd is globally associated with transcribed chromatin and activates transcription. These conclusions agree in part with our observations.

## Materials and Methods

### Construction of HeLa cell lines expressing tagged histones H2A, macroH2A1.1 and H2A.Bbd

HeLa S3 cells were grown in Dulbecco modified Eagle medium (DMEM) supplemented with 10% foetal calf serum. Human testicular seminoma cell line JKT1 was a kind gift from Dr. Saadi Khochbin and was grown in DMEM (Invitrogen, USA) supplemented with 2% sodium pyruvate and 10% FBS (Invitrogen).

The HeLa S3 cells expressing the epitope-tagged histones were generated by retroviral transduction and magnetic sorting according to [45]. Retroviral pOZ vectors contain a bicistronic transcriptional unit that allows expression of two proteins from a single transcript. This design ensures tight coupling between expression of the gene of interest and the selection marker, the interleukin-2 receptor a-chain (IL2Ra) [45]. In short, the Phoenix E cells [46], obtained from ATCC, were calcium-transfected with the pOZ.FHHC [47] vector carrying the histone ORFs, and two days after transfection the viral supernatant was used to infect Hela S3 cells stably expressing murine ecotropic receptor. Two days after retroviral transduction, the infected cells were subjected to magnetic sorting procedure using Dynabeads M-450 goat anti-mouse IgG, coupled to Mouse anti-IL2Ra antibody (Upstate Biotechnology, Inc.). The sorting was repeated 2 times and the obtained polyclonal population of cells was used for further work.

### Immunofluorescent microscopy

Cells were fixed, stained with anti-FLAG antibodies and/or antibodies against macroH2A and antibodies against H3K27Me3 (Active Motif) and observed by fluorescence microscopy. At least 100 transfected cells were inspected per each histone variant using a Zeiss Fluorescent Microscope and representative images captured.

### Western blotting

Western blotting has been carried out according to the standard protocol. The primaty antibodies against macroH2A (No. 39593, Active Motif, USA) were revealed using the ECL+ system (GE Healthcare, USA) according to manufacturer's instructions.

### qRT-PCR

The expression level of histone H2A.Bbd was determined by qRT-PCR using specific primers (sense primer 5′CTACCTCGCTGCGGTTATTG3′, antisense primer 5′GTCGTGTTGAAAAGGGTGCTC3′). 400 ng of total RNA purified via Trizol (Invitrogen) was converted into cDNA using 8 independent pools of primers (#4384791, Applied Biosystems, AB) and TaqMan microRNA Reverse transcription kit (#4366596, AB). cDNA was quantified using via qPCR using TaqMan 2× Universal PCR Master Mix.

### Isolation of DNA associated with tagged histones H2A, macroH2A1.1 and H2A.Bbd

For chromatin isolation, nuclei were prepared by incubating cells in hypotonic buffer (10 mM Tris-HCl, pH 7.5, 10 mM KCl, 1.5 mM MgCl2, 0.1% Triton X-100, 0.2 mM PMSF). After washing with 600 mM NaCl in 20 mM Tris, pH 8.0, 10% glycerol, 0.1% Tween 20, 0.2 mM PMSF, the nuclei were resuspended in nuclease digestion buffer (0.34 M sucrose, 10 mM Tris-HCl, pH 8, 3 mM MgCl2, 1 mM CaCl2, 0.2 mM PMSF) and treated with micrococcal nuclease for a desired time interval. The reaction was stopped with 4 mM EDTA. To release the undigested chromatin, the pellet was sonicated and the size of chromatin fragments (1–3 nucleosomes) was monitored by electrophoresis in a 2% agarose gel. The non-solubilized material was removed by centrifugation at 16,000 *g* for 30 min, and the supernatant was dialyzed against 300 mM KCl, 20 mM Tris-HCl, pH 8.0, 10% glycerol, 0.1% Tween 20, 0.2 mM PMSF overnight. The precipitated material was removed by centrifugation at 45,000 *g* for 30 min.

The double-tag purification was performed as described [45], except that at the second step Ni-nitrilotriacetic acid (NTA)-agarose (QIAGEN) was used. Briefly, 1 ml of chromatin solution was incubated overnight with 100 µl of the anti-FLAG agarose beads (Sigma) and washed five times with 300 mM KCl in 20 mM Tris-HCl, pH 8.0, 10% glycerol, 0.1% Tween 20, 0.2 mM PMSF and the epitope-tagged containing chromatin fragments were eluted with 100 µl of FLAG peptide (1 mg/ml FLAG peptide in 300 mM KCl in 20 mM Tris-HCl, pH 8.0, 10% glycerol, 0.1% Tween 20). Ten microliters of Ni-NTA-agarose was added to the FLAG eluate and incubated for 2 hours. The Ni-NTA beads were washed with the same washing buffer. The final elution was performed with 2 M NaCl in 20 mM Tris-HCl, pH 8.0, 10% glycerol, 0.1% Tween 20. Histone-bound DNA was purified by phenol extraction and, after amplification as recommended (Nimblegen), DNA samples were hybridized to a human genome tiling array consisting of 50 mers positioned every 100 bp along nonrepeating sequences of the selected regions of chromosomes 11, 16 and X. Raw data were collected by Nimblegen (Roche-NimbleGen, Iceland). The results of all ChIP experiments were deposited in the GEO database under the reference GSE28041.

### Analysis of the ChIP tiling array data

Signal intensity data were extracted from the scanned images of each array using Roche NimbleScan software. The ratio of the input signals for the experimental and control samples that were co-hybridised to the array was computed for each feature of the array. Then the log_2_ ratio was computed. Subsequently, the NimbleGen log_2_ ratio files have been analysed using ACME (Algorithm for Capturing Microarray Enrichment) [32], [33], written in R language and freely available through Bioconductor. ACME supports data analysis for any NimbleGen tiled array design. The software is based on the assumption that the real signal is represented by multiple probes that are genomically located close to one another, producing a neighbour effect. After loading the data into R, ACME automatically sorts probes by their genomic location. The user must then set a threshold within the distribution of the ratio measurements above which true positive signals are expected to be enriched. We have set this threshold at 0.85 or the 85^th^ percentile. To identify potential sites of enrichment, a window of user-defined size (window is of size 400, corresponding to 1–2 nucleosomes) moves stepwise along the tiled region, centering at every probe and testing if it contains a higher than expected number of probes over the defined threshold = 0.85 (chi-squared test). Single probes that yield high intensity ratios most likely represent noise and are automatically filtered out by the windowing/threshold analysis [33]. The resulting output contains treated p-values (−log_10_(p-value)) with corresponding chromosome coordinates. We have imported these results into the Integrated Genome Browser [31] for visualization. Analyses performed at other window sizes and threshold produced similar results (data not shown).

While computing p-values using ACME, independence between individual data points was violated to an unknown degree [32]. We used a permutation-based algorithm to assess the significance of the ACME p-values and to establish relevant cutoffs likely to be representative for H2A.Bbd and macroH2A1.1 binding events. The null hypothesis is that the probe label order has no effect upon the computed ACME p-values. If changing the order of the probe labels destroys the effect, then a random permutation test can be done. Hence, we have generated 40 permuted samples for each of the 2 datasets (H2A.Bbd and macroH2A1.1). We processed the ChIP-chip signal with ACME for each of the permuted datasets, and then computed the False Discovery Rates (FDR), corresponding to the ACME p-value of each probe. FDR was computed by counting the rate of permutation ACME p-values which were larger than the ACME p-value obtained on the real dataset. According to the obtained results, for p-value <0.0001 (−log_10_(p-value)>4), the FDRs were very close to zero, meaning that peaks characterized by −log_10_(p-value)>4 were not likely to be discovered by chance (Figure S4). For p-values <0.00001 (−log_10_(p-value)>5) the FDR is strictly 0. Thus, we set two 2 cutoff values, a loose one at 4 and a tight one at 5.5. All p-values situated between loose and tight cutoffs were attributed to a Marginal significance class.

### Correlation with gene expression data

Gene expression data was obtained from GEO (Gene Expression Omnibus, [34]). Dataset GDS2526 [35] contains two samples (GSM136097 and GSM136095) of gene expression profiles for HeLa cells obtained using the Affymetrix Human Genome U133 Plus 2.0 Array. We downloaded the raw intensity files and used Bioconductor's RMA utility to perform convolution background correction, quantile normalisation and a summarization based on a multi-array model fit [48]. Next, each probe was affected the average expression value between the two available microarrays. In order to represent the gene expression and ChIP-chip correspondence, we have plotted max(−log_10_(ACME-p-values)) corresponding to the following intervals: (1) Transcription start −400 bp; Transcription start+gene body, and (2) Transcription start −1000 bp; Transcription start +500 bp against the expression values (max(log_2_(Intensity_AffyProbes))) for each gene. Thus, for situations when there were several peaks in one gene, only the maximum peak was taken into consideration, implying that the plots do not really quantify the amount of sites with histone variants, but the probability that the variants are present on at least one site. When number of peaks associated with a gene has been more than one, this number is indicated to the right of the corresponding point on the plot.

## Supporting Information

Figure S1 Cellular distribution of macroH2A1.1 protein in control HeLa S3 cells and HeLa S3 cells transfected with FLAG-tagged MacroH2A1.1 gene. (**a** and **b**) control (**a**) and transfected (**b**) HeLa S3 cells immunostained with antibodies against MacroH2A1.1 and secondary antibodies conjugated with Alexa488. Nuclei were conterstained with ToPro3 to visualize DNA. The intensities of fluorescence were measured using Zeiss LSM Image Browser for 3 nuclei (graphs under the images). On the graphs the relative intensities of ToPro3 (DNA staining) and Alexa 488 (MacroH2A1.1 staining) are shown by blue and green curves. Note similar fluorescent intensities of MacroH2A1.1 in both types of measured cells. Two nuclei with higher intensity of MacroH2A1.1 in image **b** are indicated by arrows. (**c**) Distribution of recombinant macro H2A1.1 (immunostained with anti-FLAG antibodies and secondary antibodies conjugated with Cy3) and all cellular macroH2A1.1 (immunostained as in sections **a,b**) in HeLa S3 cells transfected with FLAG-tagged macroH2A1.1 gene. The intensities of fluorescence were measured using Zeiss LSM Image Browser for 3 nuclei (graphs under the images). On the graphs the relative intensities of Cy3 (FLAG-tagged macroH2A1.1 staining) and Alexa 488 (total macroH2A1.1 staining) are shown by purple and green curves Bars correspond to 10 µm.
(TIF)

Figure S2 False Discovery Rates (FDR) observed with the cutoffs set at different ACME p-Values. The graphs show results of random permutation tests carried out on the 2 datasets (H2A.Bbd and macroH2A1.1) for the selected regions of chromosome 11 and chromosome 16. For −log_10_(p-value) >4 the false discovery rate (FDR) was very close to zero and it was strictly zero for −log_10_(p-value) >5.5, therefore each peak that exceeds the value of 4 can be considered as significant (see Materials and Methods for details).
(TIF)

Figure S3 H2A.Bbd (max (−log_10_(ACME-p-values)) in potential promoter area [−1000; +500] versus gene expression levels, for genes present in the selected areas of chromosomes 11 and 16. See the text for details.
(TIF)

Figure S4 macroH2A1.1 (max (−log_10_(ACME-p-values)) in potential promoter area [−1000; +500] versus gene expression levels, for genes present in the selected areas of chromosomes 11 and 16. See the text for details.
(TIF)
