# Supplementary figures and images for: Distinct Distribution of Ectopically Expressed Histone Variants H2A.Bbd and MacroH2A in Open and Closed Chromatin Domains
Source: PLoS One. 2012 Oct 30;7(10):e47157. doi: 10.1371/journal.pone.0047157 (PMC3484066; doi:10.1371/journal.pone.0047157)

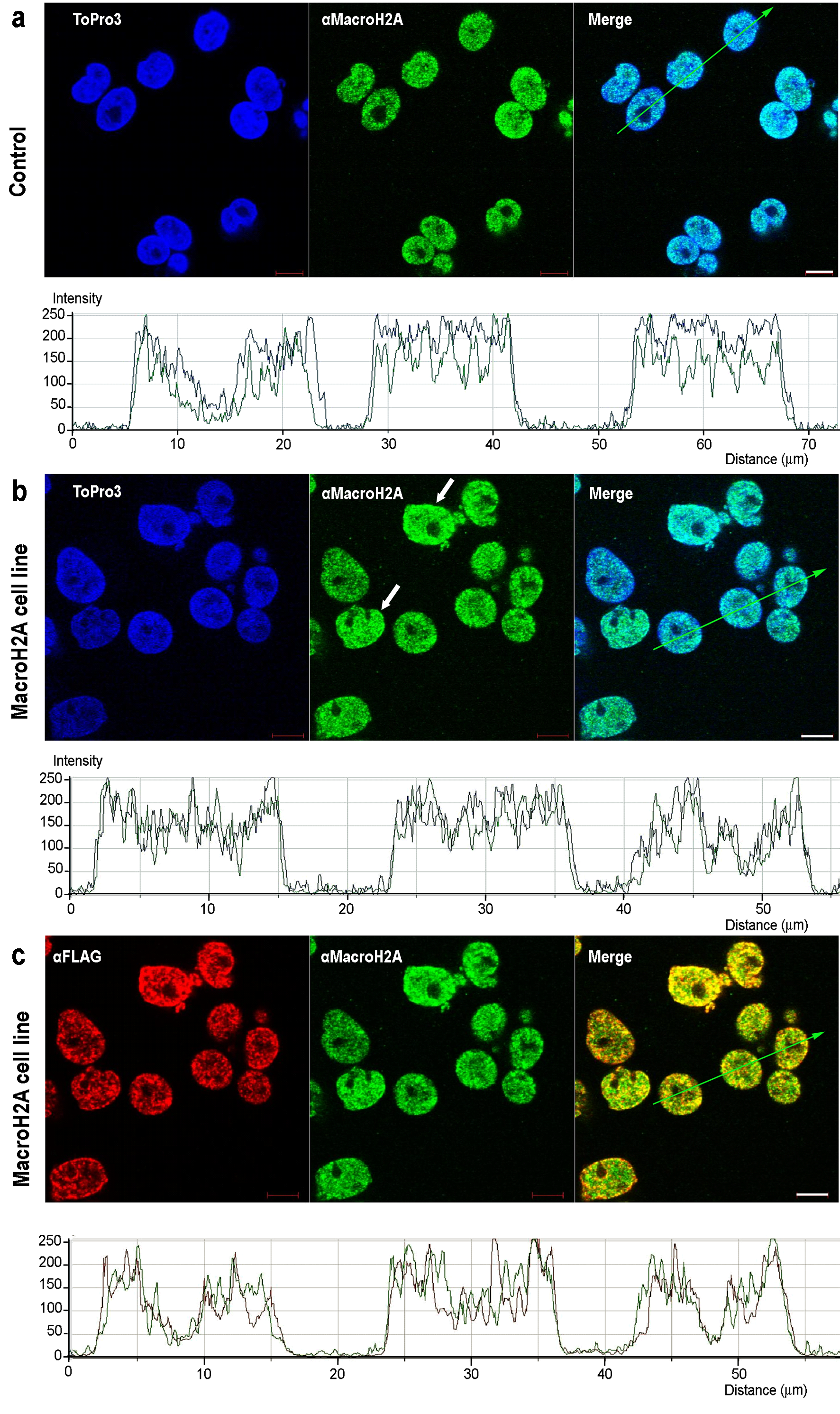

Supplement: Figure S1 — Cellular distribution of macroH2A1.1 protein in control HeLa S3 cells and HeLa S3 cells transfected with FLAG-tagged MacroH2A1.1 gene. (a and b) control (a) and transfected (b) HeLa S3 cells immunostained with antibodies against MacroH2A1.1 and secondary antibodies conjugated with Alexa488. Nuclei were conterstained with ToPro3 to visualize DNA. The intensities of fluorescence were measured using Zeiss LSM Image Browser for 3 nuclei (graphs under the images). On the graphs the relative intensities of ToPro3 (DNA staining) and Alexa 488 (MacroH2A1.1 staining) are shown by blue and green curves. Note similar fluorescent intensities of MacroH2A1.1 in both types of measured cells. Two nuclei with higher intensity of MacroH2A1.1 in image b are indicated by arrows. (c) Distribution of recombinant macro H2A1.1 (immunostained with anti-FLAG antibodies and secondary antibodies conjugated with Cy3) and all cellular macroH2A1.1 (immunostained as in sections a,b) in HeLa S3 cells transfected with FLAG-tagged macroH2A1.1 gene. The intensities of fluorescence were measured using Zeiss LSM Image Browser for 3 nuclei (graphs under the images). On the graphs the relative intensities of Cy3 (FLAG-tagged macroH2A1.1 staining) and Alexa 488 (total macroH2A1.1 staining) are shown by purple and green curves Bars correspond to 10 µm. (TIF) [file pone.0047157.s001.tif]

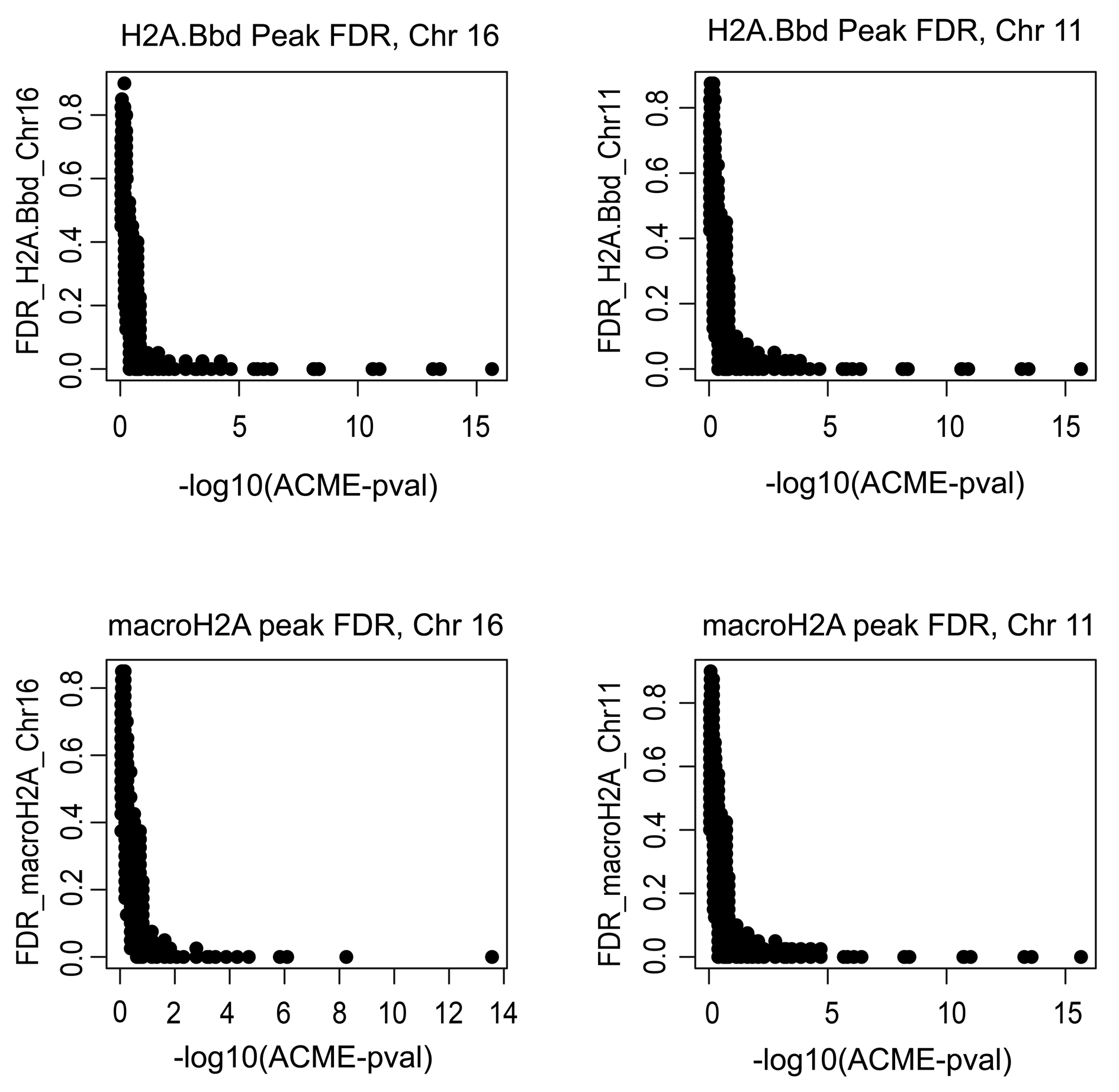

Supplement: Figure S2 — False Discovery Rates (FDR) observed with the cutoffs set at different ACME p-Values. The graphs show results of random permutation tests carried out on the 2 datasets (H2A.Bbd and macroH2A1.1) for the selected regions of chromosome 11 and chromosome 16. For −log10(p-value) >4 the false discovery rate (FDR) was very close to zero and it was strictly zero for −log10(p-value) >5.5, therefore each peak that exceeds the value of 4 can be considered as significant (see Materials and Methods for details). (TIF) [file pone.0047157.s002.tif]

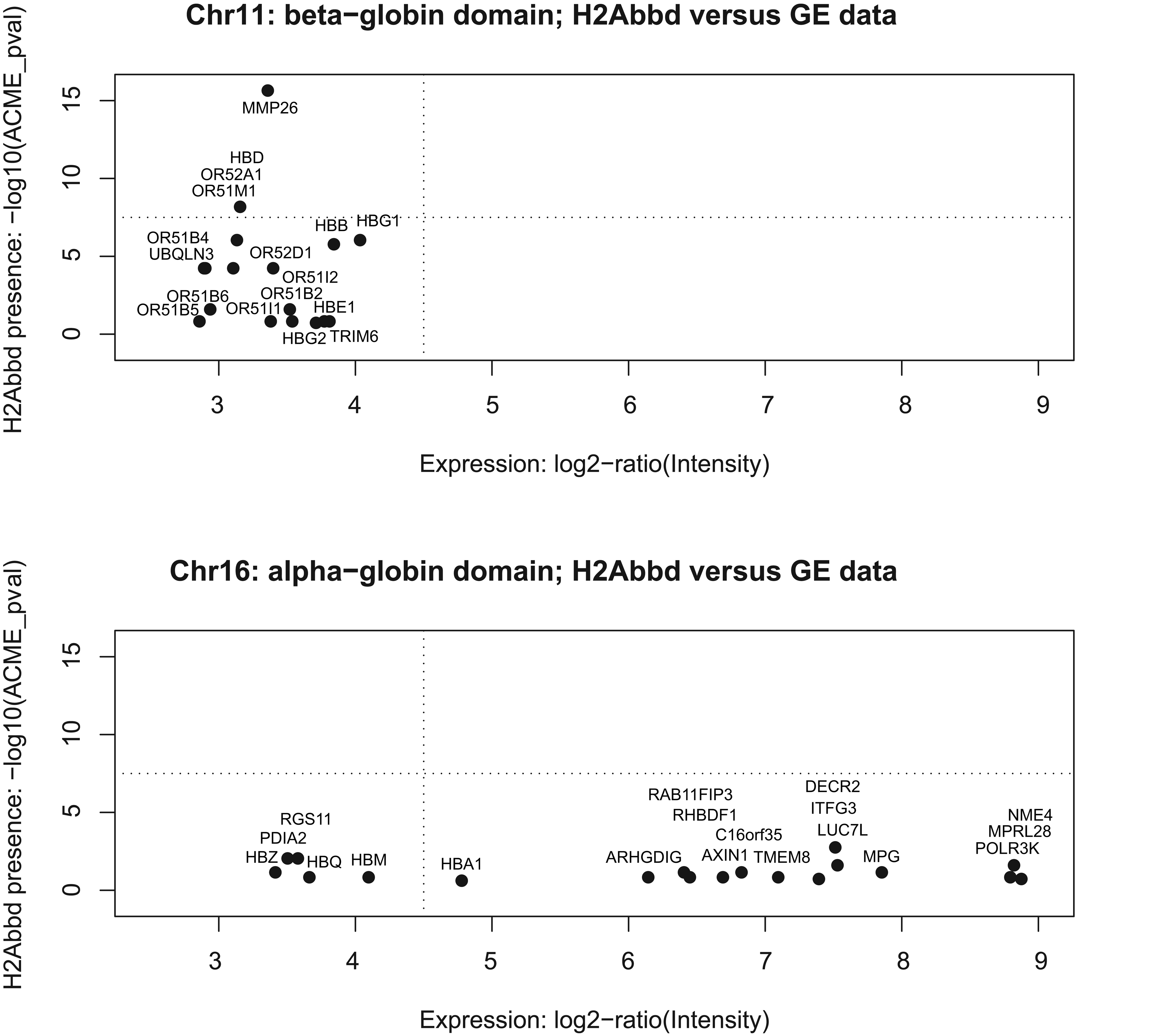

Supplement: Figure S3 — H2A.Bbd (max (−log10(ACME-p-values)) in potential promoter area [−1000; +500] versus gene expression levels, for genes present in the selected areas of chromosomes 11 and 16. See the text for details. (TIF) [file pone.0047157.s003.tif]

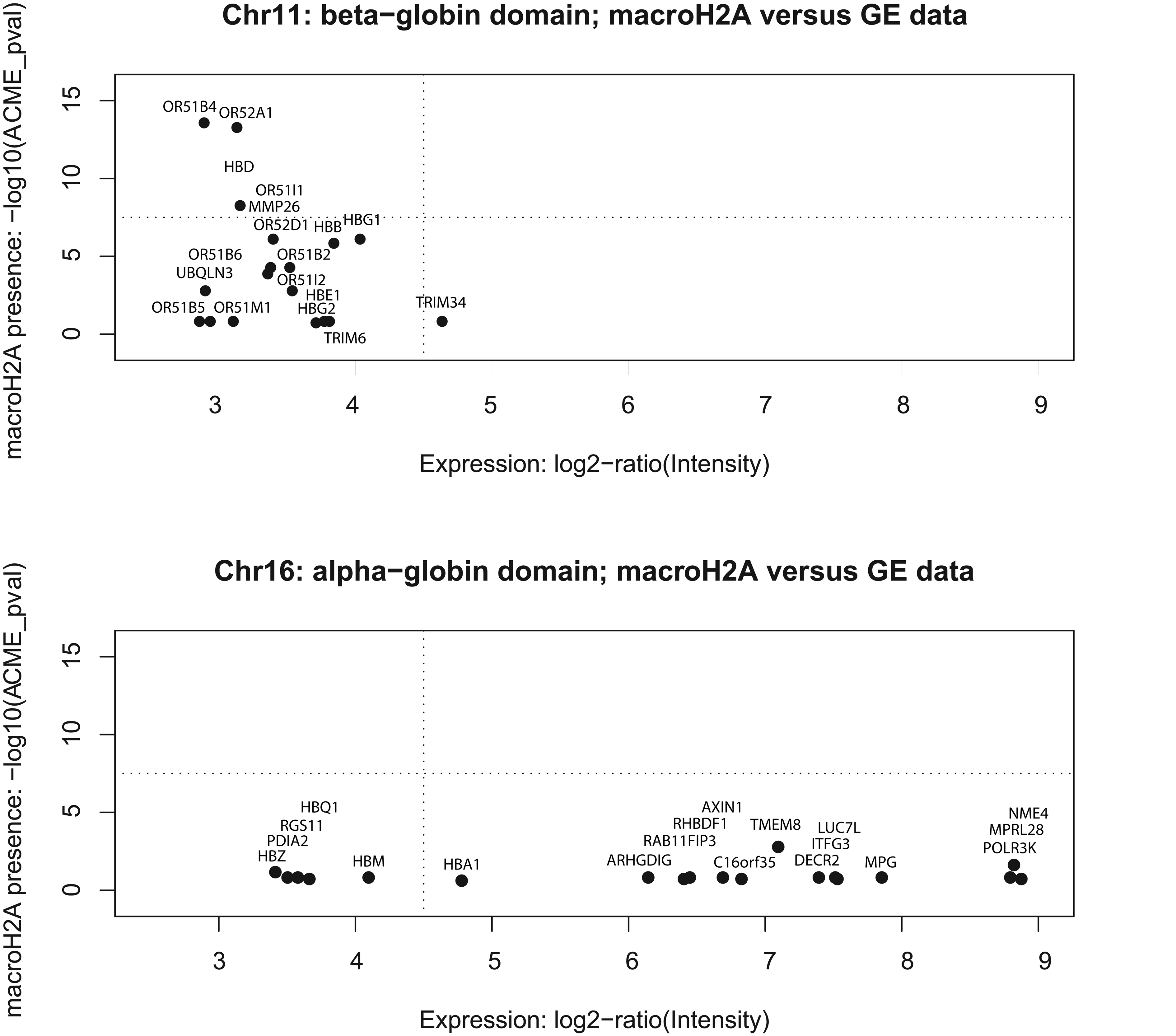

Supplement: Figure S4 — macroH2A1.1 (max (−log10(ACME-p-values)) in potential promoter area [−1000; +500] versus gene expression levels, for genes present in the selected areas of chromosomes 11 and 16. See the text for details. (TIF) [file pone.0047157.s004.tif]
